# Breeding unicorns: Developing trustworthy and scalable randomness beacons

**DOI:** 10.1371/journal.pone.0232261

**Published:** 2020-04-28

**Authors:** Samvid Dharanikota, Michael Toft Jensen, Sebastian Rom Kristensen, Mathias Sass Michno, Yvonne-Anne Pignolet, René Rydhof Hansen, Stefan Schmid

**Affiliations:** 1 Faculty of Computer Science, University of Vienna, Vienna, Austria; 2 Department of Computer Science, Aalborg University, Aalborg, Denmark; 3 DFINITY, Zurich, Switzerland; Wuhan University, CHINA

## Abstract

Randomness beacons are services that periodically emit a random number, allowing users to base decisions on the same random value without trusting anyone: ideally, the randomness beacon does not only produce unpredictable values, but is also of low computational complexity for the users, bias-resistant and publicly verifiable. Such randomness beacons can serve as an important primitive for smart contracts in a variety of contexts. This paper first presents a structured security analysis, based on which we then design, implement, and evaluate a trustworthy and efficient randomness beacon. Our approach does not require users to register or run any computationally intensive operations. We then compare different implementation and deployment options on distributed ledgers, and report on an Ethereum smart contract-based lottery using our beacon.

## 1 Introduction

A *randomness beacon* is a service emitting unpredictable random values at regular intervals, defined in 1983 by Michael O. Rabin who used it to add probabilistic security in several protocols [1]. Randomness beacons can help a group of users to agree on some random outcome, even though they do not trust each other. In particular, the main purpose of the randomness beacon is *not* to produce “better” local random numbers than, e.g., using /dev/urandom; it allows users to agree on the same random value. Randomness beacons come with many applications, e.g., in cryptographic, security, and distributed systems protocols. Example applications include generation of protocol parameters and seeding elliptic curves [2, 3], privacy preserving messaging [4–6], anonymous browsing [7–9], electronic voting and secure elections [9], gambling and lottery services, or preventing selfish mining [10–12]. Randomness beacons are considered a “tool of democracy” [2].

Not surprisingly, there is an abundance of approaches for the design of publicly-verifiable, bias-resistant and unpredictable randomness beacons. There are two main strands of beacon research with different computational requirements on the users. One type of beacons require a beacon operator which provides its users with random values. In such approaches, the beacon operator bears the main computational burden. In the second type of design, all participants are equal and share the computational complexity more equally. Depending on the application and the computational constraints of the actors involved in a use case, one or the other of the two is more appropriate. In this paper, we focus on the first case, with a powerful beacon operator offering its service to light-weight users.

While in early versions of randomness beacons of the first type, the beacon operator itself needed to be trusted (i.e., it is an unbiased third party), with obvious implications for security, recent literature has sought to design beacons where the need to trust the beacon operator is reduced or removed entirely.

In keeping with this trend, we design and implement a randomness beacon that works under the most pessimistic assumption possible: everybody (in particular, this includes the beacon operator) is secretly colluding against the user and is willing to invest money and resources towards manipulating or biasing the randomness. Specifically, we seek a design that minimizes the trust required by a user and also allows each user to decide how much they *want* to trust the beacon such that, a user will know that under self-chosen trust assumptions, the randomness has not been manipulated.

Randomness beacons are of particular interest in the context of distributed ledgers and smart contracts, steering the interaction of mutually distrusting parties. In such scenarios trustworthy randomness can speed up computations and break symmetries. Although many potential implementations and practical solutions are discussed in the literature on randomness beacons, very few actual implementations of public, general-purpose beacons have been published or made available. We describe the design and deployment options for our randomness beacon on a smart contract platform and their implications.

In summary, this paper makes the following ***contributions***. After a thorough threat analysis, we design, implement and evaluate a practical, secure, and trust-minimizing randomness beacon based on the *transparent authority* model which relies on *user input*. The design captures the requirements derived through the structured analysis of threats to a randomness beacon and builds upon the unicorn protocol devised by Lenstra and Wesolowski [2], but can be employed more generally. It allows users to send inputs and consume beacon values at any time and at low overhead without a registration procedure. Our implementation relies on parallelized computation, which minimizes the possibility of malicious operation while avoiding idle periods. Furthermore and unlike other approaches of transparent authorities, the beacon operator in our beacon design has no private information: all inputs are hashed and are released to the public in batches before the computation. The beacon also offers users to make subtle decisions on when to trust the output. Our beacon uses Merkle trees as the data structure for inputs to reduce the computation proof size. Our experiments with a first prototype demonstrate the scalability of our approach.

We further illustrate how this beacon can be deployed on distributed ledger platforms. We compare different (partial) on- and off-chain deployment options and discuss our experience and evaluation of Ethereum smart contracts for a lottery with our beacon.

To ensure reproducibility of our results as well as to facilitate follow-up work, we share our implementations on https://github.com/randomchain/randbeacon.

**Bibliographic note**. A preliminary version of this paper was presented at the IEEE Blockchain conference 2019 [13]. We extend this work with a security taxonomy and threat analysis, an extended evaluation and a more thorough survey of related work.

## 2 Basic beacon concepts and related work

Randomness beacons and related functionality have been studied intensively in the literature already, see [10–12, 14–20] for a list of but a few examples.

The two main concepts of a random beacon concern its *input* and *beacon operation*. The input describes what sources are used to calculate the beacon value, while the beacon operation describes the design of the protocol, i.e. how to collect the input, perform the computation and publish the output.

Input sources can be split into three categories. A beacon operator can use its *private source* of data to produce randomness. This potentially allows users to consume randomness of high quality at a high rate, but denies users access to inspect the process and thus requires users to trust the beacon and its randomness. It does not align with our stated security goals, since the beacon outputs cannot reliably be distinguished from carefully crafted values that appear to be random. An example of this input source model is the NIST randomness beacon, developed by the National Institute of Standards and Technology (NIST), which observes quantum mechanical effects to produce what is claimed to be high-quality randomness [14, 16]. As such, the users need to blindly trust the beacon operator, i.e., NIST in this case [21–23]. Beacons based on *publicly available sources* cover input from sources that are publicly available and which everyone can agree on the value of, e.g., financial data [18], lottery numbers [17] or bitcoin block hashes [15, 19]. The users must trust the source to be sufficiently random, which may be fine for the examples mentioned. Finally, beacons can also rely on *user input* in which a user is allowed to directly provide input to the beacon. The idea is that a user provides a value that they believe is sufficiently random. The beacon then performs an operation on the set of user-supplied inputs, yielding an output that allows all users

1. to *verify the inclusion* of their input and
2. to *verify the validity* of the computation.

If these are satisfied, the user knows that a value they trust to be random has been part of the random output generation. The computation performed by the beacon should ensure that users cannot knowingly bias the output to anyone’s disadvantage. As such, users know their input was not knowingly “counteracted” by another user.

We can distinguish between three models for beacon operation, detailed below. In the *autocratic collector* model, a beacon is run by a party which requires blind trust from the users. As such, the computation is a black box with no possibility for proof of honesty. An alternative is to use *specialized MPC* where users utilize Multi-Party Computation (MPC) to collectively produce randomness, typically from their own inputs. Given an honest majority, this type of beacon produces randomness that is not biased against the participants. Despite significant work in the field, this approach is difficult to scale to large groups since any addition or removal of a user requires a new setup phase [10, 11, 20]. This type of beacon is therefore not well-suited for public settings with vast numbers of users, but might fit in a controlled private context. Finally, in a *transparent authority* model, a single entity collects inputs and publishes them with a focus on transparency. Users can, by observing the beacon, verify that it behaves according to the protocol. This does not directly prevent Byzantine behavior, but rather makes it difficult to hide such behavior. This type also supports a wide variety of implementations, and can be scaled to a public setting. In this paper, we focus on transparent authorities and provide a scalable implementation of such a randomness beacon. One of its crucial advantages is the fact that it does not require users to register or run any computationally intensive operations.

The “zoo approach” [2], describes a protocol reminiscent of a beacon which collects data from a variety of sources before running them through a verifiable delay function called *sloth*. Sloth is a strictly sequential function which is orders of magnitude faster to inverse for verification. The time-hardness prevents last-draw attacks, as attackers have to dedicate large amounts of time to compute how to bias the output, during which new inputs can render their efforts pointless. The sloth delay function is a also key part of our randomness beacon. However, the supporting structures driving the beacon are designed differently and we analyse the security of both the protocol and the beacon operator in more detail; in particular, we assume the beacon operator can be malicious. A *unicorn* protocol is then used to combine input collection from multiple sources and then compute the output of a delay function. This protocol resembles that of the *transparent authority* beacon computation model, and is done by a single entity. Lenstra and Wesolowski suggest feeding *sloth* with an aggregation of user inputs. Furthermore, the authors present a protocol named *trx*, which utilizes the output of the unicorn protocol. While they guarantee random unpredictable outputs even if all other users are malicious, they do not explore the scenario of a malicious operator, who colludes with adversarial users. We built upon their approach and design a system that can tolerate a malicious beacon operator, while keeping the communication and computation cost for users low. Other approaches that require users to register and entail a communication complexity of *O*(*n*^2^) (e.g., [20]) and rely on the assumption of at most 1/3 Byzantine nodes in the system. In contrast, in our system users need to interact with the beacon operator two times, once to submit their input and once to obtain the output. If they want to verify their input has been considered and the output is valid, the complexity is in *O*(log *n*) due to the Merkle tree structure.

There exist other verifiable delay functions beyond sloth. Bünz et al. [19] evaluate the computation and verification of delay functions based on modular square roots and the hashing functions Keccak-256 (SHA3) and SHA-256. Subsequently, [24] formalized the notion and present functions that achieve an exponential gap between evaluation and verification time. Note that sloth could be replaced by these functions in our implementation and most likely achieve better performance. Since the focus of this paper is on more general system aspects, we omit an evaluation of these functions in this paper.

## 3 Threat analysis

We start by performing a *threat analysis*, considering possible threats to a generic randomness beacon in order to understand the threat environment.

### 3.1 DREAD analysis

Our analysis assumes the *user input* model of input as well as a beacon based on the *transparent authority* model. In our setting, randomness is the fundamental resource that adversaries would attempt to threaten and control. Thus we consider the *availability* and *integrity* of the randomness beacon output to be the primary targets for attackers. We furthermore distinguish between *insiders* and *outsiders*: an insider is anyone with the capabilities of the beacon operator (for example the beacon operator itself), but for all intents and purposes may as well be anyone gaining insider access to the beacon, e.g., by hijacking it. Because the beacon operator should not be trusted either, we see no reason to distinguish between a legitimate beacon operator, a malicious beacon operator, or an adversary maliciously acquiring access to the inside of the beacon. In this context, an outsider is anyone who can only influence the beacon operation from the outside network, and thus does not have inside access.

In order to structure our threat analysis, we employ the well-known DREAD framework (with a slight modification commonly used) [25]. In this framework, potential threats are evaluated against five criteria and given a score on a simple scale 1 (indicating a *low* score) over 2 (indicating a *medium* score) to 3 (indicating a *high* score). The individual scores are based on a *qualitative* assessment by the analyst. Threats are then assigned a final DREAD score, comprising the sum of the individual scores, yielding a ranking of threats in which those with high (final) scores are those that are considered the most dangerous, i.e., attacks that can cause a lot of damage and are (relatively) easy to carry out score higher than threats that are unlikely or very difficult to realize. It is important to note that these scores are qualitative and abstract and thus mainly useful for *relative* ranking of threats and should not be assigned any particular quantitative interpretation. The five individual evaluation criteria are as follows

1. Damage: How harmful would such an attack be? This includes considerations of breach of safety, loss of privacy, financial loss.
2. Reproducibility: How easy is it to reproduce such an attack? This includes robustness of exploits (e.g. portability of attack across platforms and platform variations), (financial) cost of performing an attack.
3. Exploitability: How much (or rather, how *little*) work is required to launch the attack (with the 3 being the least amount of work)? This includes the amount of preparation for an attack, degree of specialisation needed for an attack, e.g., custom designed attacks.
4. Affected users: How many users will be impacted? This is used as a rough measure of the impact of the attack.
5. Discoverability: How easy is it to discover the vulnerability? This is an estimate of the amount of work and resources necessary to detect a vulnerability. Note that for some critical applications, it is recommended to assume the highest score for discoverability to avoid “rewarding” security by obscurity. Indeed, most of the threats detailed below, have high discoverability, as they are mostly obvious, but may be hard or expensive to implement.

Table 1 summarizes the findings of our threat analysis and in the following we will briefly describe these in more detail, using a shorthand notation for indicating the (numerical) results of our DREAD analysis, e.g., “Input Flooding $\begin{array}{llllll} \text{D} & \text{R} & \text{E} & \text{A} & \text{D} & \Sigma \\ 2 & 3 & 2 & 3 & 3 & 13 \end{array}$”, where each of the capital letters refer to the corresponding DREAD category and *Σ* refers to the total DREAD score, where anything equal to or above 12 is considered *high* risk.

**Table 1:** Attacks and their DREAD score.

	Insider	Outsider
**Threats to availability**	Beacon shutdown (12) Withholding output (12)	Input flooding (13) Eclipse beacon (10) Eclipse select users (8)
**Threats to integrity**	Input manipulation (14) Leak output (14) Emit false output (11)	Input biasing (12) Output degradation (13) Man in the middle (11) Cyptography exploit (10)

#### 3.1.1 Threats to availability

We start by describing some of the potential threats to *availability*. Such threats are often hard to protect against and can have serious consequences for users and applications that depend on timely computation of random numbers.

- **Shutdown** $\begin{array}{llllll} \text{D} & \text{R} & \text{E} & \text{A} & \text{D} & \Sigma \\ 2 & 2 & 2 & 3 & 3 & 12 \end{array}$ A malicious beacon operator can shut the beacon down, completely denying availability. This threat is impossible to prevent for a beacon run by a single operator, although the beacon operator will likely not get away with it.
- **Withholding Output** $\begin{array}{llllll} \text{D} & \text{R} & \text{E} & \text{A} & \text{D} & \Sigma \\ 2 & 2 & 2 & 3 & 3 & 12 \end{array}$ The operator can withhold outputs that are not favorable to its interests. This threat is also quite significant and may be difficult to detect/prove.
- **Input Flooding** $\begin{array}{llllll} \text{D} & \text{R} & \text{E} & \text{A} & \text{D} & \Sigma \\ 2 & 3 & 2 & 3 & 3 & 13 \end{array}$ Outsiders can overwhelm the beacon with inputs to prevent other users from contributing their own input, or simply perform a Denial-of-Service (DoS) attack on the beacon server. This is a serious threat as the attack is quite easy to execute.
- **Eclipsing (Select) Users** $\begin{array}{llllll} \text{D} & \text{R} & \text{E} & \text{A} & \text{D} & \Sigma \\ 2 & 1 & 1 & 1 & 3 & 8 \end{array}$ An outsider can deny select users from accessing the beacon to provide input or receive output. This is arguably a smaller threat, as it is difficult to prevent a determined party from accessing the beacon, and such an eclipse would still only affect that party.
- **Eclipsing the Beacon** $\begin{array}{llllll} \text{D} & \text{R} & \text{E} & \text{A} & \text{D} & \Sigma \\ 2 & 1 & 1 & 3 & 3 & 10 \end{array}$ An outsider can deny all users from providing input or receiving the output by infiltrating the inbound and outbound connection to the beacon. This may be a difficult attack to execute, but if successful the outsider can potentially eclipse the beacon from all users.

#### 3.1.2 Threats to integrity

These threats can be far more damaging than threats to availability if not detected: Where availability is binary and users obviously cannot use a missing output, successful integrity attacks provide an output, that appears legitimate, but is biased. We consider using a biased output the worst thing for any user, which makes these threats critical.

**Input Biasing** $\begin{array}{llllll} \text{D} & \text{R} & \text{E} & \text{A} & \text{D} & \Sigma \\ 3 & 2 & 2 & 3 & 2 & 12 \end{array}$ An outsider can provide input that biases the output to their benefit. In this attack the outsider constructs an input such that it affects the output in a known way despite other users contributing input later. If the outsider has the capability of providing the last input, it may launch a *last-draw attack*. This is a severe threat to the beacon, as the adversary is able to freely manipulate the output with their input, and violates the unpredictability of the random number. The attack can be executed by anyone with access to the input collectors given that they have the ability to pre-compute outputs.
**Input Manipulation** $\begin{array}{llllll} \text{D} & \text{R} & \text{E} & \text{A} & \text{D} & \Sigma \\ 3 & 3 & 2 & 3 & 3 & 14 \end{array}$ The operator can manipulate the input to bias the output of the beacon. It can also selectively exclude inputs from certain users to deny them availability. This threat is severe as the operator may manipulate the inputs, in a way that cannot be detected. It is also easy for any operator capable of pre-computing the output, and affects the randomness given to all users.
**Output Degradation** $\begin{array}{llllll} \text{D} & \text{R} & \text{E} & \text{A} & \text{D} & \Sigma \\ 2 & 3 & 3 & 2 & 3 & 14 \end{array}$ Adversaries can supply “bad” input to reduce the quality of the output. This is also a serious threat as it will affect the quality of randomness provided to all users, a randomness which may not even be usable. In addition, it is easy to do given access to the input collectors, and could even happen by accident.
**Man in the Middle** $\begin{array}{llllll} \text{D} & \text{R} & \text{E} & \text{A} & \text{D} & \Sigma \\ 3 & 1 & 1 & 3 & 3 & 11 \end{array}$ Adversaries can intercept and change data sent between user and beacon. This threat could be significantly damaging but also extremely hard to execute for adversaries. Due to the nature of beacons we recommend using them when you need to agree on some random number—thus, to intercept and manipulate inputs and outputs, the adversary would have to distribute the manipulated number to all users, as they would otherwise disagree on the numbers, leading to the manipulation being discovered.
**Emitting False Output** $\begin{array}{llllll} \text{D} & \text{R} & \text{E} & \text{A} & \text{D} & \Sigma \\ 2 & 1 & 2 & 3 & 3 & 11 \end{array}$ A malicious operator can output false results of the computation that benefit him. While this is technically a threat to the integrity of the beacon, the effects should be similar to those of a withholding attack. This is due to the fact that simply publishing false output would rapidly be discovered in a transparent authority beacon, making the output unusable, but also removing any faith in the operator.
**Leaking Output** $\begin{array}{llllll} \text{D} & \text{R} & \text{E} & \text{A} & \text{D} & \Sigma \\ 3 & 3 & 2 & 3 & 3 & 14 \end{array}$ The operator can give access to the output earlier to some parties than others—potentially selling early access. This threat can be quite severe, as we do not know how early access can be granted compared to when the randomness is used. It also violates the unpredictability property of the beacon, and is easily executable for any malicious operator of the beacon. In the worst case it would affect all users.
**Cryptography Exploit** $\begin{array}{llllll} \text{D} & \text{R} & \text{E} & \text{A} & \text{D} & \Sigma \\ 3 & 2 & 1 & 3 & 1 & 10 \end{array}$ Weaknesses or exploits may exist in the cryptographic techniques that protect the beacon. While we estimate it will be hard to find such exploits, hence the low discoverability score, they would likely be relatively easy to apply once found, and would affect all users. In this case one might also consider the effect quantum computers would have on the use of cryptography, which could also threaten the beacon.

## 4 Design

This section describes our beacon design, aiming to mitigate the threats identified above.

### 4.1 Requirements

This section lists the requirements for a randomness beacon suitable for our security goals and the threats that exist towards beacons. We decided on using the *transparent authority* type of beacon, which requires a high level of transparency, and as such we build requirements on top of that.

- *Transparent Operation* Users should be able to oversee that the beacon operates according to the protocol and thus catch any deviations from it. Being able to verify whether their own input has been used, allows users to determine whether they should trust the output. Furthermore, users should be able to repeat the process on their own computers as a means of verification. This also requires the process to be deterministic. However, the output should still be unpredictable, even to the beacon operator.
- *Open and Secure Protocol* Anyone should be able to easily contribute to the beacon protocol to influence the random generation. There should be no requirements imposed on users to limit their contribution rate besides denial of service protection. The protocol should be secure meaning that even if only a single user is honest, the output is still unpredictable.
- *Timely Publishing* The protocol should enforce that input, output, and any data needed for verification of an output is published as soon as possible to make the beacon more transparent. By having a requirement of timeliness at the protocol level, we restrict the time a malicious operator has available to diverge from protocol before users will suspect them.
  Giving users all the tools to replicate and oversee the process makes it difficult for adversaries to covertly manipulate the beacon to their benefit, and allows users to complete output computation themselves if the beacon stalls. This in turn mitigates one of the greatest threats from the operator, input manipulation (see below). A beacon that does not reveal which inputs were used before publishing the output will essentially be admitting that they picked the inputs to bias the output.
  We should note that despite having this property the beacon does not guarantee outputs on any specific wall-clock time, e.g., at 12:00:00, 12:01:00, and 12:02:00. Instead, it will output as soon as possible after each period of input collection. Barring any attacks, this will provide a regular stream of outputs.
- *Practicality* Scalability of all components is important to be suitable for many use cases. Therefore, it should scale to at least several thousand users contributing with user input in every output. It will be beneficial to allow different channels for input and output, both to make the beacon easier to access for users, but also to make it resilient to having any single channel attacked. We also consider fault tolerance a valuable property to have, and having multiple channels still allows users to input if one fails.

### 4.2 Service oriented architecture

To meet the requirements of modular input and output and fault tolerance, we use a service oriented architecture (SOA) in the beacon design. This architecture splits the system into services that each serve a single purpose. Communication between services is done according to a well-defined protocol. In addition to scalability, a service oriented architecture provides loose coupling and further simplifies fault tolerance since individual services can easily be replicated.

**Fig 1:**
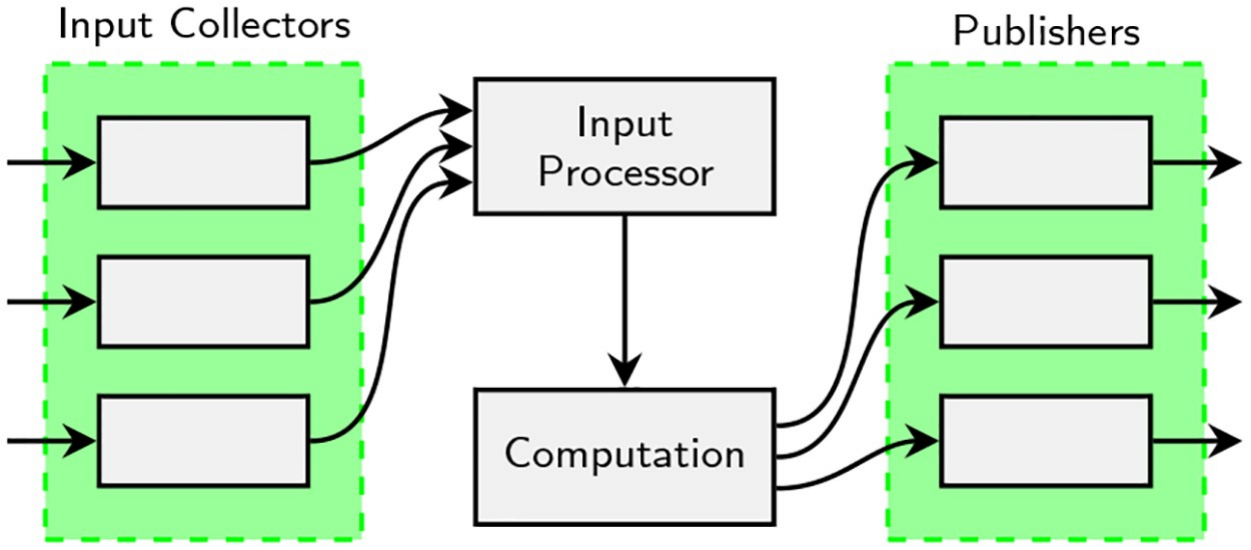
An abstract beacon architecture based on services. Solid boxes illustrate services and arrows represent data flow.

A randomness beacon designed as a service oriented architecture consists of a number of input collector services that collect input from many different sources. An input processor service aggregates the input from all collectors and forwards it to the computation service, which commits to the aggregated input and runs the computation to generate an output. Finally, various publisher services publish the commitment, output, and any relevant proofs to different outlets. Fig 1 illustrates this architecture.

### 4.3 Security design

A major security concern is the operator’s ability to predict or manipulate the output. Our solution for this problem is to ensure that each published output is paired with a commitment which can be used in the verification of the beacon. As a novel design decision, the commitment must contain all data required for the computation and all inputs. This is different from the approach based on a trusted operator where the operator can work with the inputs [2]: In Unicorn, the beacon operator commits to an input that is revealed when publishing the next output [2]. In order to replace the operator and implement it in a distributed way, commits are useful.

The transparency allows any party, as a strategic choice, to compute the randomness alongside the beacon operator. It ensures that the operator cannot cause much damage by withholding output or by deciding not to open a traditional, e.g., hashed commitment. This hence reduces the “value” of the output: depending on the timing, it is less attractive to leak output, i.e., sell early access to the output, as everyone can just compute it. Put differently, while this does not prevent the operator of performing a withholding attack, it minimizes the effects of it. Note that the user has a choice here: depending on the user’s preferences, it can choose to invest more resources and compute the output from the commitment and obtain the valid output itself; as a different design choice, the user may be fine with learning the output slightly later but efficiently.

To further decrease the possibilities of the operator trying different commitments before releasing them, we use a *verifiable delay function*. Delay functions can be seen as black box functions that require a given amount of time to run and are inherently sequential, meaning they cannot benefit from parallel execution. It ensures that the output cannot be instantly computed, and that the operator cannot try more than one commitment before running out of time. As such, the operator is unable to perform the input manipulation attack in a meaningful way. In order to avoid excessive computation by users performing verification, delay functions used in randomness beacons should be hard to compute and easy to verify, i.e., they must be *asymmetrically hard*. The operator is of course able to exclude or change output, but not in a way that knowingly benefits anyone because the effect of the manipulation is hidden behind the delay function.

The delay function also protects against *last-draw attacks*, where an adversary attempts to bias the output by crafting an input to produce favorable randomness. The adversary needs to compute the result of adding a specific input as the last input. Delay functions make this significantly more difficult to attempt due to the time needed to compute the result. Given a delay function that takes five minutes to complete, an adversary must dedicate five minutes of processor time to any given input he attempts to use. This means he must dedicate large amounts of resources to perform any significant number of attempts, and more importantly if a single input is added to the beacon within that five minute period, all of his work will be null, and he will be forced to restart.

We use the delay function *sloth* [2]. As mentioned earlier, there is no secret input to the delay function in our design. Note that in [2], a different attacker model is used. More precisely, the beacon operator wanted to safeguard against adversaries trying to manipulate the outcome. In this work, we consider the beacon operator as potentially malicious. Therefore, we proposed that the operator produced a commitment to a set of inputs, while also revealing the inputs. This effectively means that anyone can calculate the delay function, and potentially be faster than the operator. We deemed that by having the operator include a secret input, to prevent anyone from computing the outcome before himself, the trust implications are too severe, as a user would have to trust that the operator did not try multiple secret values in parallel and chose the most beneficial outcome. In our design, an adversary may know the outcome earlier than an honest participant that waits for the beacon operator to announce it. However, the adversary cannot bias the outcome, as long as there is at least one honest party. This can hence be considered a design tradeoff, as everyone can learn about the output immediately by investing the resources to compute the value once the commits are known.

**Rational trust assumptions**. In our approach we want to push beyond the need for honest operators and naïve users. To achieve this we extend the work of [2] to quantify trusting the beacon and determine thresholds for reasonable behavior when using delay functions. This provides a measure of rational trust, where users decide for themselves if what they observe is adequate.

We present a property which, if satisfied, means a user can trust that the beacon operator is not capable of fooling them. This property is true if the user determines that nobody is able to compute the delay function in the time between the users input and the user receiving the beacon’s commitment to the input for the delay function. This can be condensed to

$$\begin{array}{c} t_{\text{COMMITMENT}}-t_{\text{INPUT}}\;<\;T_{\text{DELAY}\;\text{FUNCTION}} \end{array}$$

where *t*_INPUT_ is the time when the user sent the input, *t*_COMMITMENT_ is when the user received the commitment, and *T*_DELAY FUNCTION_ is the fastest computation of the delay function. So for users to be more likely to trust a beacon, the time between sending the input and receiving the commitment must be significantly smaller than the time between the commitment and the output. In fact, it must be smaller than the shortest time the user thinks the operator could compute the delay function.

An example could be that a user believes that the world’s fastest computer can compute the delay function in two minutes. In this case the users can trust the output if they see a commit to a set of inputs containing their input within two minutes of their input having been sent. This relation between the time taken to compute the delay function and the time before a commitment is seen allows users to flexibly adjust their willingness to trust the outcome has not been biased against them.

A similar threshold is also described by [2], where they advise a ratio of no more than one fifth of the computation time spent collecting inputs. In their paper, the authors furthermore state that participants will always try to minimize the time between their input and the commitment. We see this as potentially problematic, since such behavior can create congestion in the system, which might result in some inputs not being used in the intended output computation. This means that users whose inputs were not included cannot trust the output of the given beacon iteration.

### 4.4 Parallelization

**Fig 2:**
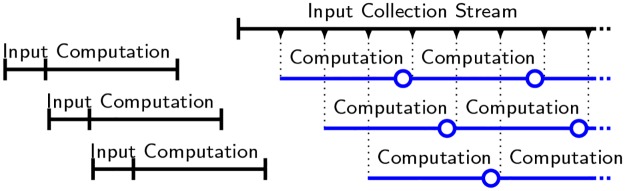
Parallelized beacon protocol, with offset input collection (left) and stream input collection (right). Beacon output is published after computation, last vertical lines (left) and circles (right).

Taking all this into consideration we present a beacon operation protocol which can be adjusted to increase or decrease the ratio and thereby the limit for probabilistic trust. The operation must be sequential which means that we must collect input before computing the delay function. Accordingly, we propose to decouple input collection and computation, and to parallelize the latter. This means that several delay functions run in parallel, but are offset in time and on different input, illustrated in Fig 2 (left).

We observe that no input collection is run in parallel nor overlapping, which resembles a constant stream of input collection. In addition, the computation resources can be reused for future beacon computations, thereby eliminating the need for spinning up new computation services, as depicted in Fig 2 (left), where the beacon would output at each circle shown in the diagram.

#### 4.4.1 Number of computation nodes

The number of computation nodes at least required in this fashion is given by the duration of the delay function divided by the duration of input collection. As computational times typically vary slightly, this can cause the beacon output to be more skewed compared to the initial output frequency. To remedy this, we account for possible extra time *δ* for each delay function. In this case,

$$\begin{array}{c} \text{Number}\;\text{of}\;\text{Nodes}=\lceil \frac{T_{\text{DELAY}}\;\text{FUNCTION}+\delta }{T_{\text{INPUT}}\;\text{COLLECTION}}\rceil \end{array}$$

If, for example, the delay function is guaranteed to finish at most 2 minutes later than the expected time of 10 minutes, i.e., a worst-case time of 12 minutes, and the input collection is 2 minutes, 6 nodes in total are necessary to guarantee a node is always ready every 2 minutes.

## 5 Prototype implementation

In this section we give a brief overview of the implementation of our beacon design. Our prototype has been implemented mainly using Python 3 with a few subcomponents written in C for performance.

The message passing infrastructure of our SOA is implemented using the *ZeroMQ* framework for asynchronous message passing and concurrency (available at http://zeromq.org). We can directly employ the “publish/subscribe” pattern provided by *ZeroMQ* between computation nodes and publisher. This pattern handles the message routing based on subscription prefixes, resulting in less traffic on the network. Furthermore, the fan-in for input collectors is implemented with a “push/pull” socket pair which ensures fair operation, thereby avoiding starvation of components. Lastly, *ZeroMQ* guarantees atomic delivery of messages, which means that we can assume all parts of a message or none at all.

To avoid implementing heavy service discovery functionality and to simplify configuration, we deploy proxies at key points in the pipeline: one between input collectors and the input processor and one between computation and publishers.

### 5.1 System interface

As previously mentioned, the system boundaries, i.e. where users and the outside world interacts with the beacon, are handled by input collectors and publishers. We implement these and the surrounding infrastructure, as well as vertical scaling if the load becomes too high on a single component.

To limit the space of potential messages and message sizes passed around inside of our system, we sanitize the user inputs by hashing them at the entry point with the SHA512 hashing algorithm. Realistically, allowing *any* input could be seen as an invitation by some users to post messages or even files, e.g. illegal or inappropriate content. Our choice of hashing at entry point will mitigate this. Given a substantial number of users, receiving and hashing inputs may become a costly affair performance-wise. Fortunately, the state of an input collector is only relevant to a single input request, meaning that scaling and even distributing across many machines is a trivial task. When we hash an input, as a convenience we return the hashed input as a response. As such, they will later be able to confirm that their hashed input was used in the output of the beacon. To allow users to verify correct hashing, the hashing algorithm should be made publicly known. Currently we use the SHA512 hashing algorithm since its digest size is 64 bytes, which gives us reasonably sized messages flowing through the system, while still having 2^512^ possible different values. It could be argued that the 32 bytes of SHA256 are more than enough for any use case. However, SHA512 is actually roughly 1.5 times faster than SHA256 on a 64-bit CPU [26]. Therefore, we see no reason to limit the possibilities to 2^256^, since we do not expect 512 bits per input to be too much data. We implement the system such that the chosen hashing algorithm can be configured at beacon start.

### 5.2 Combining inputs

One of the most important tasks of our implementation is to combine the (hashes of the) collected input both as a preparation for the computation phase, but also to derive commitment data that can be verified by users. As a novel contribution, our implementation uses a *Merkle tree* for this purpose. A Merkle tree is a special binary tree where the value of each node is the hash of the concatenation of its two children; here the leaf nodes are the hashes of user inputs and the root node is then the condensed output.

Merkle trees as commitment data allows third-party applications to provide verification, since the inclusion of a given leaf node in a Merkle tree can be verified by providing all siblings to the nodes on the path up to the root. This greatly limits the amount of data which the user needs to fetch and process to *O*(log *n*) where *n* is the number of leaf nodes in a Merkle tree. The commitment data consist of an ordered list of the leaf nodes.

Another property of the Merkle tree is that, like hashing a concatenation of all collected inputs, each leaf node equally affects the root node, due to the diffusion property of the hashing algorithm. This means that any change to the set of inputs changes the root node in the Merkle tree.

### 5.3 Parallel computation

As discussed, we need parallel and time offset computations in the beacon. This is achieved by letting the input processor handle the scheduling of computations: The beacon is configured to process inputs at a lower bounded interval, which means that the input processor will send work at fixed times, given an available computation component. It should be noted that if no such computational component is available, the input processor will just continue collecting input. If no computation service becomes available within a given threshold, the input processor will give a warning to the system operator.

The worker announcements and subsequent work assignments are facilitated with *ZeroMQ*’s “router/dealer” socket pair which allows asynchronous addressed messaging. When a computational node connects to the input processor it sends a READY message, receives an OK, and proceeds to wait for incoming work; this process, accompanied by what follows inside the computational node, can be seen in Algorithm 1. The input processor then keeps track of each announced worker, and when the time comes, sends condensed processing output and commitment data to the next free worker.

If the worker does not acknowledge the work with an OK response, the inputs are reprocessed, and the next free worker is assigned. This cycle continues until a worker accepts the work, while new incoming inputs are included in each reprocessing of inputs. Having duplex communication between the input processor and the computation nodes is a practical compromise between a strict pipeline pattern and a monolithic input processor/computation node.

**Algorithm 1 Specification of computational node outlining the communication pattern with the input processor**.

1 **procedure** Initialization()

2  connectTo(input processor, publishing proxy)

3 **end procedure**

4 **procedure** MainLoop()

5  **repeat**

6   sendToInputProcessor(READY)

7   **if** OK received before timeout **then**

8    *W* ← receiveWork() ▹ blocking call

9    **if** *W* is valid **then**

10     sendToInputProcessor(OK)

11     startComputation(*W*_input_)

12     sendToPublish(*W*_commit_)

13     **wait for** computation to finish

14     *C* ← collectComputationResult()

15     sendToPublish(*C*_output_, *C*_proof_)

16    **else**

17     sendMessage(ERROR)

18    **end if**

19   **else**

20    **continue**

21   **end if**

22  **until** the end of time

23 **end proceodure**

### 5.4 Delay function

For the computation phase we implement a delay function based on *sloth*, the function proposed to be used in the unicorn protocol [2]. The general idea behind *sloth* is to iterate through modular square root permutations of a large prime number and thereby construct a time hard algorithm, while containing a trapdoor for fast reversal, i.e., verification. Essentially, the verification calculates squares of the output from the computation. When implementing delay functions in systems that rely on their time guarantees, it is important to focus on performance, since an obvious yet undeployed optimization of execution time would compromise the “time hardness” of the algorithm. We implement *sloth* as a Python module with a C-extension for the actual algorithm. In the C-extension the GNU MP library https://gmplib.org/ is used to perform integer arithmetics with large numbers.

## 6 Performance evaluation

We conducted several experiments to explore potential system bottlenecks to gauge reasonable throughput. We also investigate our chosen delay function *sloth* and different configurations of it.

All experiments are executed on a server with an *Intel Core i7-2600* CPU, which runs at 3.40 GHz. The server has four cores and can hence run 4 simultaneous sloth computations. We use *SHA512* as the hashing algorithm in both the Merkle tree and in the *sloth* delay function.

### 6.1 Bottleneck analysis

We examine the potential bottlenecks which require the most effort to scale horizontally: the proxies and the input processor.

#### 6.1.1 Proxies

As discussed, our beacon contains two proxies. While the *forward* proxy between computation and publishers is unproblematic in any real world randomness beacon deployment (it only forwards outputs, commitments, and proofs), the *stream* proxy situated between input collectors and input processors may become a bottleneck, as it has to handle a constant stream of input messages. Recall that this proxy facilitates fan-in and fan-out pipelining with fair message distribution using a round-robin strategy. Hence, we test the throughput of the proxy in different configurations of input collectors and input processors. For simplicity and benchmark consistency, we utilize “dummy” components for this. The input collectors are referred to as *pushers* and fan in at the proxy, while the input processors are called *pullers* and fan out. In the tests we transmit messages which resemble those of an actual beacon in size, i.e. 64 bytes of application data plus any *ZeroMQ* packaging; in this case one byte which serves as a flags field, and one byte to denote the message length.

**Fig 3:**
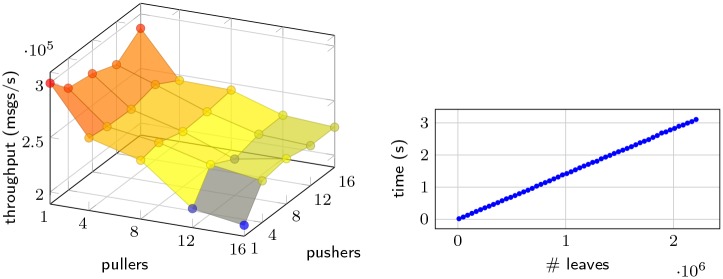
Configuration effect on throughput of messages in proxy. (Left) 64 bytes message throughput per second of *stream* proxy, with different numbers of pullers and pushers. (Right) Correlation between number of leaves and the time it takes to build a Merkle tree with those leaves.

In Fig 3 (left) we see how the aforementioned different configurations affect the throughput of messages in the proxy. Firstly, every combination shows a throughput of at least 200k messages per second: likely sufficient even for popular real world beacons. It is the scenario of one pusher to sixteen pullers that results in the lowest throughput, which can be caused by the overhead of the fair message distribution enforcement. However, as we add pushers at sixteen pullers, a slight increase in throughput can be seen, suggesting that fair distribution is easier with more suppliers.

Another observation we can make from Fig 3 (left) is that increasing the number of pushers does not affect the throughput as much as adding pullers does. This illustrates that fan-out is a considerably more expensive task than fan-in—a fortunate fact, since a deployment of our beacon most likely will consist of remarkably more pushers than pullers.

We can conclude that the proxies in our system are unlikely to be bottlenecks, and we should rather look further down the pipeline for issues; hence we next examine the input processor.

#### 6.1.2 Input processor—Building merkle trees

The most expensive task in our input processor is building the Merkle tree. This task is done periodically when it is time to compute a new random output. It is critical that this computation is fast, as this step can extend the time between the last seen input and publishing the commitment. As such, we examine how the number of leaves, i.e. inputs, affects the building time of the Merkle tree. In Fig 3 (right), a linear growth in build time is seen as a factor of the number of leaves. The growth is slow and is negligible in our beacon. Well over 2M leaves are needed to result in a build time over 3*s*.

Admittedly, the build time could be a problem if significantly many inputs are used. However, in this case one might reimplement the input processor in a more performant language than Python, e.g., C. In addition, the construction of Merkle trees is trivially parallelized. Our evaluation results presented here do not take advantage of this fact. Thus building subtrees in multiple processes and merging them to form the final tree provide a significant speed-up with a factor close to the number of available CPU cores.

### 6.2 Sensitivity analysis of *sloth*

The computation and verification time of the delay function, *sloth*, can be configured by adjusting two parameters: *(1)* the number of bits of the prime number used in the computation; and *(2)* the number of times to iterate through the permutation process of said prime.

**Fig 4:**
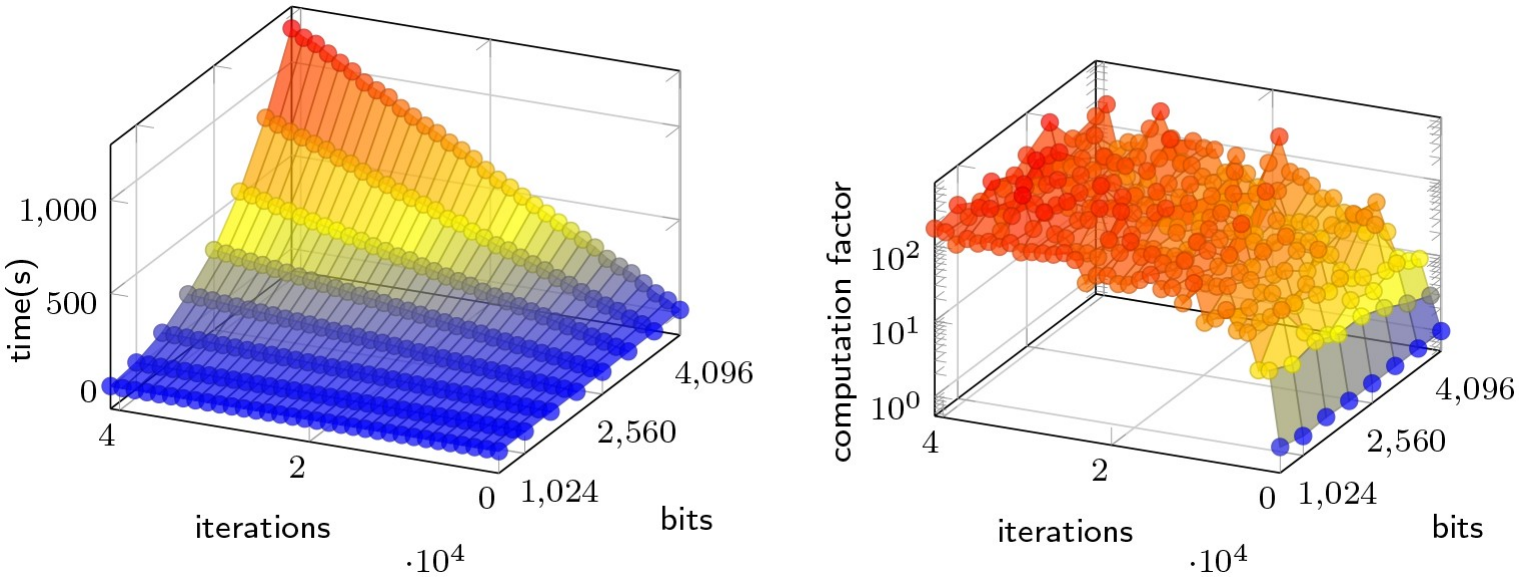
Execution time of *sloth* computation and verification with different parameters. (Left) Bits and iterations vs time of computation. (Right) Computation time vs verification time with the logarithmic *z*-axis.

To evaluate the *sloth* delay function and its sensitivity on the parameters, we run a series of tests of the algorithm. During the tests we sample multiple rounds with random inputs and take the average. Fig 4 (left) illustrates the correlation between the two parameters, and the time it subsequently takes to do a computation with a given combination of bits and iterations. An increase in the number of bits used for the prime number results in an exponential growth of the computation time, while an increase in number of iterations cause a linear growth.

While computation time is important for the delay function, another significant metric is verification time—especially in relation to the computation time. Fig 4 (right) illustrates this relationship, where the *z*-axis shows how many more times it takes to compute the output relative to how long it takes to verify. Although the data is more scattered than in the previous figure, we see a trend where the growth of this factor levels out just above one hundred. This means that in configurations with more than roughly 3K iterations, the computation time is always more than two orders of magnitude larger than the verification time.

We also observe that the number of bits does not affect the factor except for some irregularities in the data. These irregularities are caused by the extra time it potentially can take to initially find the prime number; an operation which can vary in time depending on how close the numeric representation of the hashed input string is to a prime. Since larger primes (given by number of bits) can be more difficult to find, the data fluctuates more at larger number of bits.

## 7 Blockchain applications and implementations

The big promise of blockchains is to facilitate business interactions between mutually distrusting parties, ranging from virtual currency transfers to smart contracts, enabling the trusted execution of arbitrary code. More precisely, the tamperproof nature of an append-only distributed ledger realized by a blockchain protocol, forms the basis for platforms to run smart contracts. In essence, a smart contract is a piece of code stored at an address on the ledger. Sending a message to this address triggers the execution of the code with the arguments in the message, and the resulting state is stored on the ledger. A blockchain consensus protocol ensures that all parties agree on the operations and their sequence, as well as the resulting state, despite the presence of malicious participants and the lack of trust.

Since randomness beacons let parties that do not trust each other base decisions on a trusted source of randomness, we study the implications of an implementation of our beacon in a blockchain environment. Smart contracts between mutually distrusting parties can benefit from unbiased trustworthy randomness to speed up computations and break symmetries, e.g., in games. Since openness to any users is key in our design, an implementation providing randomness on a public permissionless blockchain, which allows any interested entity to participate, makes most sense. We first compare different implementation options and then report on a blockchain-based implementation of a lottery application using our beacon.

### 7.1 Design choices

There are essentially two options for the implementation:

- The actual beacon operator is run as a smart contract.
- The beacon operator runs separately from the blockchain, but publishes some of its artifacts as part of smart contract on the blockchain.

While a fully blockchain-based solution offers benefits in terms of decentralization (no single point of trust/failure, more robust to attacks including DoS, …), this solution is costly as each computation in the smart contract consumes virtual currency. This ties the beacon into the monetary incentive structures that dictate smart contract behavior. Due to the high cost, this option requires many users to compensate for the large on-chain computation cost.

The second implementation option offers several variants with different trade-offs. The verification can, for example, be done either on-chain in a smart contract, or by each interested user on their own. This has the advantage that expensive on-chain computations are avoided. Which artifacts and computation are on-chain and what parameter size are appropriate is an application-specific tradeoff between security and costs.

E.g., storage cost is proportional to the size of data stored on-chain, thus storing (parts of the) data off-chain, e.g., one blockchain-backed distributed hashtables or IPFS, may provide a solution with some guarantees that tampering with the commitment and output will be detected, yet with a lower price tag.

The time necessary for a proposed transaction to be in a block that can be considered immutable may be highly variable depending on the nature of the underlying blockchain. Since the time interval between submitting an input and receiving a commitment from the beacon operator is the basis of trust for a user, the blockchain latency must be taken into account when configuring the delay function. Moreover the variability of the blockchain latency may tarnish the trust assumption of users. In addition, since parts of the beacon would still be off-chain, those parts will depend on an operator and are vulnerable to DoS attacks.

We study a *lottery* application based on our beacon, and to compare different implementations more systematically, we consider the following 3 players:

- *Owner*—Runs the lottery (e.g., smart contract owner)
- *User*—Takes part in the lottery by sending a small payment to the lottery smart contract
- *Beacon*—Beacon operator that provides a random value for the drawing of a lucky winner

The main goal of the lottery owner is to shave off some of the users’ participation payments as a reward. In other words, not all of the user payments are given to the lucky winner, some of it is transferred to the lottery owner. Users only want to participate in a lottery when they have a reason to trust that the random value provided by the beacon is not biased, i.e., if they sent some input to the beacon to influence the generated random value and received a commitment within their trust time bound (on or off-chain).

We consider the following implementations, ordered by increasing on-chain smart contract complexity:

- *Maximum off-chain (OFF)*: In this implementation all beacon-related logic is off-chain: only the lottery logic is on-chain. The users send their inputs to the beacon off-chain and obtain the commitment off-chain. Thereafter they send the lottery payment to the smart contract. When the off-chain beacon value computation has finished, the lottery smart contract fetches the value with an oracle. Using this value, it then determines the winner of the lottery. Users can verify if the beacon matches the commitment and complain off-chain and decide not to trust this beacon in the future. This has no influence on the outcome of the current draw of the lottery. Both the owner and the winning user receive rewards through the execution of the smart contract, while the beacon operator is remunerated off-chain.
- *Adding beacon incentive and on-chain commitment (ITV)*: To allow the beacon operator to be compensated with the smart contract, the following changes can be made to the simple contract proposed above. In a first step, the beacon publishes its public key and a nonce and locks some funds in the smart contract. Users send their input to the beacon off-chain and once they see their input included in the commitment, they send (i) the Merkle tree root signed by the beacon operator together with the nonce locked earlier and (ii) their participation payment to the smart contract. In this scenario, the beacon operator submits the next beacon value to the smart contract directly or it is fetched with an oracle call. The verification of the correct execution of sloth on the Merkle root is performed on-chain. The beacon loses its locked funds if verification fails. If the verification succeeds, the owner, beacon and winner receive rewards.
- *On-chain inputs (INP)*: This version moves the input inclusion verification done by the user in the previous versions to the smart contract. In this case the user can send their input on-chain together with the lottery payment and it is then up to the beacon operator to send the corresponding commitment in time to avoid losing its locked funds. Thus the user does not have to worry about the commitment after selecting an input. Verification and reward distribution is analogous to ITV. To reduce the cost for on-chain memory and computation, a sequential commit representation is advantageous in this and the following variant.
- *Optimistic (OPT)*: Since verification is costly and needs to be paid by the smart contract owner, the beacon operator and the users, another option is to add a *complaint phase* instead of carrying out the verification computation for every draw. In this case, an entity can submit evidence within a certain time frame to the smart contract that shows that the beacon value has not been computed correctly. Upon the successful verification of the evidence, this entity then receives part of the beacon funds currently stored in the contract, the beacon loses its funds and all users get reimbursed an equal fraction of the remaining beacon funds and their lottery fees. If the complaint phase expires without such evidence being presented, the value is assumed to be valid and the winner, owner and beacon operator are rewarded correspondingly.

The different variants as well as their advantages and disadvantages are summarized in Table 2.

**Table 2:** Lottery implementation options using the transparent randomness beacon.

	Delay Function on-chain	Delay Function off-chain, growing contract complexity			
Step\Version	Full on-chain beacon	OFF	ITV	INP	OPT
**Preprocessing**	-	-	Beacon locks fund and nonce on-chain (to be released if not enough users participate within a certain time frame)	Like ITV	Like ITV
**User Input**	On-chain, together with lottery fee payment	Off-chain	Off-chain	On-chain, together with lottery fee payment	Like INP
**Beacon Commitment**	Not necessary	Off-chain. After users see their input committed in time, they send lottery fee payment	Users send Merkle root obtained off-chain (with beacon signature on root and nonce) with lottery fee payment to contract	Commitment is stored on-chain, if delivered timely and including all inputs in the commitment, else users are refunded and beacon loses fund	Like INP
**Beacon Computation**	On-chain	Off-chain, independent of lottery	Off-chain, after commitment is stored on-chain.	Like ITV	Like ITV
**Beacon Output**	On-chain	Store beacon value on-chain	Like OFF	Like OFF	Like OFF
**Post-processing**	-	User verify beacon and complain off-chain, may decide to not trust this lottery in the future (no influence on outcome of this draw)	On-chain verification. If verification is unsuccessful, beacon forfeits funds and users get lottery fees back	Like ITV	If evidence submitted by user, on-chain verification, if successful, users receive beacon funds and lottery fees, beacon forfeits funds
**Reward**	Owner and winning user receive rewards	Owner and winning user receive rewards	Owner, beacon and winning user receive rewards	Like ITV	Like ITV
**Pros**	Users do not have to worry about verification	Simple to implement, low gas consumption	Beacon compensated for its service	Beacon compensated, user only needs to interact with the contract	Beacon compensated, verification only executed on chain if someone complains
**Cons**	Requires many users to offset the on-chain computation cost, simpler and cheaper solutions without delay functions are possible for this scenario	Owner and user must know and adhere to timing of beacon, trust stems from incentives to repeat lottery execution. Beacon operator remunerated off-chain.	User interacts with off-chain beacon operator and smart contract. All honest users submit the same data. Verification executed on-chain for every draw	Verification executed on-chain for every draw, even though the beacon would typically be incentivised to be honest in this scenario	User must execute verification off-chain fast enough to react within the complaint window

### 7.2 Implementation and evaluation

We have implemented an Ethereum smart contract for the OFF and ITV models (INP and OPT are very similar to ITV from an implementation point of view) on a private test network and analyzed the gas costs for the implementations. In both the cases, the smart contract uses an oracle service to obtain the necessary data from the beacon.

**Fig 5:**
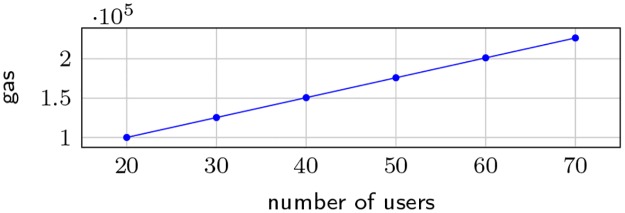
OFF gas consumption for different number of users.

Fig 5 shows the gas costs for fetching the value from the beacon and drawing a winner for different number of users in the lottery, for the OFF model. As expected, we observe a linear increase of the gas cost with number of users.

The main implementation difference between the OFF and the other variants is the fact that in the later, the verification computation can be done on-chain. Thus we compute the gas requirements for the verification (modular squaring for sloth verification) in isolation. Using delay functions that require modular squaring for verification in smart contracts is discouraged [19], owing to the high gas consumption. But the addition of a ‘pre-compiled’ contract to perform modular exponentiation as a part of EIP198 [27] significantly reduces the gas cost required to perform verification. The gas needed for modular exponentiation can be calculated based on the formula given in [27].

Table 3 shows the gas requirements for sloth verification performed for different sizes of witness, prime modulus and number of iterations. The values in the last row of the table show that for the largest evaluated witness and modulus sizes, the sloth verification cost amounts to around 5 times the cost for the rest of the smart contract with 70 users. For ITV and INP the lottery smart contract owner must set the participation fee high enough to be able to make a profit despite the verification cost. In the OPT variant, the verification computations are only executed on chain if someone submits a complaint. Thus with OPT, the owner can set a much lower participation fee as long as the locked funds by the beacon can cover the bounty and the computation cost of a successfully verified complaint.

**Table 3:** Verification gas cost for different parameter sizes.

Size of Witness (bits)	Size of Prime Modulus (bits)	Iterations	Gas
512	512	1024	159,129
1024	1024	1024	517,171
512	512	2048	368,844
1024	1024	2048	1,198,745

Note that in addition to the increase due to sloth verification computation, the amount gas required for parsing, preprocessing and validating beacon inputs, commitments, output and proof parameters including their signatures on chain has to be considered. Parsing and preprocessing can be done in multiple ways (e.g., by making multiple calls to the oracle to obtain each value individually, or making a single call and parse the returned data on-chain, and so on). It also depends on how the beacon values are encoded when sent to the contract. In addition to this, the gas costs to use the oracle service depends on the amount of data fetched. However, this part of the gas cost is dominated by far by the verification cost, so we do not report on these numbers.

### 7.3 Discussion

When using a blockchain to run (parts of) a randomness beacon, the incentive structure of all involved parties needs to be considered in a security analysis, which may include miners in public permissionless blockchains. As an example, for the trust assumption of everyone being against the user, the user would have to mine blocks to guarantee interaction with the beacon, which is a steep requirement.

We also note that using smart contracts interacting with an off-chain beacon, a beacon can also be used on a deeper level of a distributed ledger, namely as a means to speed up consensus with shared randomness. If all the members in a distributed environment trust and agree on the random value generated by the beacon, it can be used to select leaders, committees and/or rank block proposals in an otherwise trust-lacking blockchain scenario. Recent consensus algorithms leverage this idea [28, 29] with MPC beacon generation. If and how a transparent authority beacon can be applied in this context is an interesting open question.

## 8 DREAD robustness analysis

Before concluding, we revisit the threats a randomness beacon is exposed to and discuss how our proposed solution addresses them. Regarding the availability of a randomness beacon we identified the following threats: beacon shutdown, withholding output, input flooding, beacon and user eclipsing. In our design, the beacon operator’s role is to provide a service on behalf of the users, yet each of the users could replace the beacon. Thus a beacon shutdown or withholding attack is more an inconvenience than a severe threat once the input has been submitted. If the beacon or part of it is implemented on a smart contract platform, as proposed in the previous setting, the availability of the chosen platform is crucial for the availability of the beacon. With respect to input flooding, the stream proxies mitigates this threat to some extent as it separates the computation from the input processing and state-of-the-art load balancing and DOS prevention measures can be implemented for them. When implementing the input collection part of the beacon on-chain (option INP, OPT or full), the DOS resistance of the blockchain platform is inherited, which also holds for the eclipsing attacks.

Input manipulation, output degradation, man-in-the-middle, false or leaking output and cryptographic exploits threaten the integrity of randomness beacons, The integrity of our solution relies on cryptographic assumptions and thus input manipulation, output degradation and biasing are only possible if the assumptions do not hold or if the design and implementation of the cryptographic primitives contain bugs that can be exploited. State-of-the-art man in the middle prevention mechanisms should be used for crucial applications (not implemented in our version, since this is not the focus of this paper). Since the beacon output can be verified, false output can be detected. In the case of a blockchain implementation according to the options ITV, INP, OPT false output can be punished with a forfeited deposit, while a correct full on-chain implementation guarantees a correct output. Leaking output to interested parties earlier is possible, yet the value of it is questionable, since every party could compute it itself if willing to carry the cost.

## 9 Conclusion

We designed, implemented and evaluated a randomness beacon with sensible guarantees for any single user; i.e. given their random input to the beacon, they can easily and rapidly verify the computation, and decide if they deem it trustworthy. Our implementation allows all users to run the delay function in parallel or instead of beacon operator, thus mitigating the effect of a (maliciously or inadvertent) output withholding attack. Our beacon is attractive for applications based on smart contracts and distributed ledgers with minimal trust assumptions, illustrated with an Ethereum lottery application.
